# Risk factors for insulin resistance related to polycystic ovarian syndrome in Iranian population

**DOI:** 10.1038/s41598-023-37513-2

**Published:** 2023-06-24

**Authors:** Asieh Mansour, Maryam Mirahmad, Mohammad Reza Mohajeri-Tehrani, Mahdieh Jamalizadeh, Sedigheh Hosseinimousa, Fatemeh Rashidi, Pooria Asili, Sayed Mahmoud Sajjadi-Jazi

**Affiliations:** 1 Endocrinology and Metabolism Research Center, Endocrinology and Metabolism Clinical Sciences Institute, Tehran University of Medical Sciences, Tehran, Iran; 2 Endocrinology and Metabolic Research Center, Institute of Basic and Clinic Physiology Science and Department of Internal Medicine, Kerman University of Medical Science, Kerman, Iran; 3 Department of Obstetrics and Gynecology, Infertility Unit, Shariati Hospital, Tehran University of Medical Sciences, Tehran, Iran; 4 School of Medicine, Tehran University of Medical Sciences, Tehran, Iran

**Keywords:** Polycystic ovary syndrome, Metabolic syndrome

## Abstract

Polycystic ovary syndrome (PCOS) has significant metabolic sequelae linked to insulin resistance. This study aimed to compare clinical, metabolic, and hormonal characteristics of PCOS women with and without insulin resistance. The second aim was to compare the clinico-biochemical profiles of the various PCOS phenotypes. In this cross-sectional secondary analysis, we combined the baseline data from two separate randomized controlled trials (RCTs) in women diagnosed with PCOS. PCOS patients were categorized into the four Rotterdam PCOS phenotypes according to the presence of at least two criteria of oligomenorrhea/anovulation (O), hyperandrogenism (H), and polycystic ovary morphology (P): O–H–P, H–P, O–H, and O–P. Participants were categorized into two groups according to the homeostasis model assessment index of insulin resistance (HOMA-IR) levels: < 3.46, and ≥ 3.46. The correlation between the HOMA-IR and biometric, clinical, and biochemical variables was assessed in normal weight (BMI < 25) and overweight/obese (BMI ≥ 25) PCOS women. Then, the association between PCOS phenotypes and insulin resistance was investigated using logistic regression analysis. A total of 125 PCOS patients aged 18–40 years were included in the present study. Based on our results, the HOMA-IR index was positively correlated with diastolic blood pressure, free androgen index, and triglycerides levels; and negatively correlated with sex hormone-binding globulin in overweight/obese PCOS women. In addition, the HOMA-IR index was found to be positively correlated with alanine transaminase and negatively correlated with diastolic blood pressure in normal weight PCOS women. Moreover, individuals with O–H–P phenotype (odds ratio [OR] 2.52, 95% confidence interval [CI] 1.02–6.24) had about two-fold increased risk of insulin resistance. In conclusion, the full-blown PCOS (O–H–P) phenotype has an increased risk of insulin resistance. Accordingly, phenotype division may help physicians to predict adverse metabolic outcomes.

## Introduction

Polycystic ovary syndrome (PCOS) is one of the most prevalent endocrine disorders in females, affecting 10–15% of reproductive-age women worldwide^1^. Heterogeneous by nature, PCOS involves a variety of signs and symptoms of ovulatory dysfunction and androgen excess. There is a close connection between PCOS and insulin resistance, abdominal adiposity, hyperinsulinemia, and metabolic complications of these conditions^2^. The most common metabolic feature of PCOS, insulin resistance and compensatory hyperinsulinemia, affects about 35–80% of PCOS women^3^. A combination of chronic anovulation and high androgen levels correlates with insulin resistance and a higher risk of cardiovascular complications like dyslipidemia, impaired glucose tolerance, diabetes mellitus, and metabolic syndrome^4^. According to the Rotterdam criteria, the diagnosis of PCOS requires the presence of at least two of the following three features: ovulatory dysfunction, clinical and/or biochemical hyperandrogenism, and polycystic ovarian morphology^5^. Based on combinations of these three classic manifestations, PCOS women can be categorized into four main phenotypes.

Nevertheless, the question as to whether metabolic features are the same amongst all phenotypes of PCOS has yet to be addressed. Several studies have suggested that PCOS phenotypes involving oligomenorrhea or hyperandrogenemia portend a higher risk of metabolic disorders^6,7^, while some studies reported that the risk of metabolic complications does not differ among the various phenotypes, and is only related to obesity^8,9^.

Firstly, this study aimed to make a comparison between PCOS patients with and without insulin resistance, and then compare four different phenotypes of PCOS in terms of clinical and biochemical parameters.

## Methods

### Study design and subjects

In this cross-sectional secondary analysis, we combined the baseline data from two separate randomized controlled trials (RCTs) in women diagnosed with PCOS^10,11^. The two studies were randomized, prospective, placebo-controlled double-blind trials performed in Tehran, Iran, from August 2017 to May 2019. RCTs aimed to evaluate the efficacy of natural compounds (Resveratrol/Oligopin) on the hormonal and metabolic features of women diagnosed with PCOS. The protocols of both studies are available at the Iranian Registry of Clinical Trials (irct.ir) with the identifier numbers IRCT20140406017139N3 and IRCT2017061917139N2. In order to compare the hormonal parameters in insulin-resistant and non-insulin-resistant groups, the total sample size was estimated at 128 patients, using G*Power software (version 3.1.9.4) with the alpha of 0.05, power of 80% (β = 0.2) and medium effect size of 0.5.

### Inclusion criteria

Participants were included if they were diagnosed with PCOS and aged 18–40 years. PCOS was diagnosed using Rotterdam criteria^12^, by the presence of at least two of the following three conditions: (a) oligomenorrhea (menstrual cycles > 35 days)/amenorrhea (no menses for 3–6 months or longer), (b) clinical (acne, hirsutism and/or androgenic hair loss) and/or biochemical hyperandrogenism (defined by total testosterone > 70 ng/dl), (c) polycystic ovarian morphology (PCOM) on the ultrasound exam (presence of ≥ 12 follicles measuring 2–9 mm and/or ovarian volume ≥ 10 ml in at least one ovary). Based on the presence of the three criteria participants were divided into the following groups: O–H–P (i.e. presence of all three criteria including oligomenorrhea/amenorrhea (O), hyperandrogenism (H), and PCOM (P), H–P, O–H, and O–P.

### Exclusion criteria

Exclusion criteria were as follows: Breastfeeding, pregnancy, use of medications known to affect metabolism and/or ovarian function (e.g., metformin or oral contraceptives) for at least one month before the screening, history of diabetes, acromegaly, Cushing's disease, or any condition that mimics features of PCOS (e.g., non-classical congenital adrenal hyperplasia, hyperprolactinemia, or thyroid disorders).

### Measurements and assays

At the baseline visit, included participants underwent a physical examination and height, as well as systolic and diastolic blood pressure were measured. Hypertension was defined as systolic blood pressure ≥ 140 mmHg, diastolic blood pressure ≥ 90 mmHg and/or using anti-hypertensive drugs^13^. In addition, the waist circumference was measured halfway between the lowest rib and the iliac crest. Body composition and weight were assessed using the body impedance analyzer (BIA) (Tanita, Japan). Body mass index (BMI) was calculated based on the following formula: BMI = weight (kg)/height (m^2^). Participants were divided into two groups according to their BMI (normal weight [BMI < 25]; overweight/obese [BMI ≥ 25]). Acne score was assessed in four grades, as previously described elsewhere^14^. The score of hirsutism was evaluated using the Ferriman-Gallwey score system^15^.

Trans-abdominal ultrasonography was performed by experienced radiologists. Ovarian volume and a total number of antral follicles (2–9 mm in diameter) were measured using a 3 to 5.5 MHz curvilinear probe (acuson s2000, Siemens Medical Solutions, USA).

Blood samples were collected after overnight fasting and stored at − 20 °C until the analysis. Sex hormone binding globulin (SHBG) was determined by enzyme-linked immunosorbent assay (ELISA) kits (Demeditec, Germany) with the inter-assay coefficient of variation (CV) of 5.2%. The free androgen index (FAI) was determined by the equation FAI = total testosterone (ng/mL) × 3.47/SHBG (nmol/L)^16^. Luteinizing hormone (LH), follicle-stimulating hormone (FSH), testosterone, dehydroepiandrosterone (DHEA), C-peptide, and fasting insulin levels were measured by the ELISA kits (Monobind Inc. Lake Forest, California, USA) with the inter-assay CVs of 4.9%, 4.8%, 5.1%, 2.9%, and 3.8%, respectively. Concentrations of the fasting blood sugar (FBS), serum triglycerides, total cholesterol, high-density lipoprotein cholesterol (HDL), high-sensitivity C-reactive protein (hs-CRP), alanine transaminase (ALT), aspartate transaminase (AST), and creatinine (Cr) were determined by an auto-analyzer (Cobas c 311, Roche Diagnostics, Risch-Rotkreuz, Switzerland). Hemoglobin A1c (HbA1c) concentration was measured using a high-performance liquid chromatography analyzer (Tosoh, Tokyo, Japan). Friedewald formula was used to calculate low-density lipoprotein cholesterol (LDL) levels. The insulin resistance was estimated by homeostasis model assessment index of insulin resistance (HOMA-IR) with the following formula: HOMA-IR = FBS (mg/dL) × fasting insulin (μIu/mL)/405^17^. The HOMA-IR cut-off value of 3.46 was used to determine insulin resistance in PCOS patients^18^.

### Statistical analysis

Statistical analysis was conducted using the SPSS software (SPSS Inc., Chicago, IL, USA). Chi-squared test was employed to analyze trends in categorical variables. The Shapiro–Wilk test was employed to assess the normality of continuous variables. Nonparametric tests (Mann–Whitney *U* and Kruskal–Wallis tests) were used if the statistical assumptions of normality were violated. For normally distributed variables, independent sample *t*-test and one-way analysis of variance (ANOVA) were used for comparisons between means as appropriate. In case of significant differences in the ANOVA or Kruskal–Wallis test, the Bonferroni post hoc test was used for pairwise comparison.

The correlation of HOMA-IR with clinical and biochemical variables was investigated using Spearman correlation. Finally, the data were analyzed using the backward stepwise selection model with age, H–P, O–H, O–P, and O–H–P variables to estimate the odds of insulin resistance by different PCOS phenotypes. A p-value less than 0.05 was considered to be significant.

### Ethical considerations

The study was conducted in accordance with the Declaration of Helsinki and its subsequent revisions, and was approved by the ethics committee of the Tehran University of Medical Sciences (IR.TUMS.MEDICINE.REC.1399.1058). Informed consent was obtained from all study participants.

## Results

One hundred twenty-five women were included in the present analysis. Table 1 depicts the demographic, clinical, and biochemical characteristics of PCOS patients, who were divided into two distinct groups: (i) 96 (76.8%) participants with normal HOMA-IR values (< 3.46) and (ii) 29 (23.2%) participants with abnormal HOMA-IR values (≥ 3.46). The study participants were young reproductive-aged women with a median age of 28 years and a mean BMI of 27.15 ± 5.90 (kg/m^2^). The proportion of participants who reported hair loss was significantly higher in PCOS women with normal HOMA-IR compared with those with abnormal HOMA-IR (p = 0.033). PCOS women with abnormal HOMA-IR index were found to have significantly higher mean levels of BMI (p < 0.001), and anthropometric parameters including body fat percent (p < 0.001), fat mass (p < 0.001), fat-free mass (FFM) (p = 0.024), and waist circumference (p < 0.001) compared with those with normal HOMA-IR index. Furthermore, the median hs-CRP, fasting insulin, C-peptide, FBS, and triglyceride levels were significantly higher in participants with abnormal HOMA-IR values. There was also an association between FAI and SHBG levels with HOMA-IR index. Participants with abnormal HOMA-IR were shown to have higher levels of FAI (p = 0.047) and lower levels of SHBG (p = 0.003) compared with PCOS women with normal HOMA-IR index.

**Table 1:** Demographic, biochemical, and anthropometric characteristics of patients and comparison between the two groups according to HOMA-IR cutoff value of 3.46.

Variables	Total (n = 125)	HOMA-IR < 3.46 (n = 96)	HOMA-IR ≥ 3.46 (n = 29)	p-value
Age (years)^a^	28.00 (22.00–32.00)	27.50 (22.00–32.00)	29.00 (23.50–34.50)	0.256
Irregular menstruation, yes^b^	104.00 (83.20)	78.00 (81.30)	26.00 (89.70)	0.289
Hair loss, yes^b^	88.00 (70.40)	63.00 (65.60)	25 (86.20)	**0.033**
Acne score^a^	2.00 (0–2.00)	2.00 (0–2.00)	2.00 (0–2.00)	0.769
Hirsutism score^c^	10.69 ± 6.12	10.92 ± 5.84	9.96 ± 7.030	0.466
BMI (kg/m^2^)^c^	27.15 ± 5.90	25.96 ± 5.080	30.99 ± 6.80	** < 0.001**
Fat percent (%)^a^	35.70 (30.20–41.60)	34.55 (29.42–39.22)	41.9 (35.50–48.15)	** < 0.001**
Fat mass (kg)^a^	24.20 (18.20–32.10)	22.05 (17.32–28.77)	33.30 (24.20–40.95)	** < 0.001**
FFM (kg)^a^	42.80 (40.30–46.70)	42.20 (39.50–46.12)	44.20 (41.80–47.45)	**0.024**
Waist circumference (cm)^a^	91.00 (82.87–100.00)	89 (81–96.12)	100.5 (94.25–108.00)	** < 0.001**
Hypertension, yes^b^	15 (12.00)	9 (9.40)	6 (20.70)	0.112
FSH (mIU/mL)^a^	5.60 (4.20–6.80)	5.65 (4.12–6.60)	5.60 (4.20–7.40)	0.490
LH (mIU/mL)^a^	9.40 (4.40–17.15)	9.00 (4.02–16.45)	12.80 (4.65–18.05)	0.265
LH/FSH ratio^a^	1.82 (0.92–3.22)	1.75 (0.82–3.07)	2.10 (1.20–3.34)	0.357
Testosterone (ng/mL)^a^	0.40 (0.30–0.60)	0.45 (0.32–0.60)	0.40 (0.30–0.60)	0.534
FAI^a^	3.37 (1.86–6.88)	3.04 (1.73–6.45)	5.98 (2.35–8.21)	**0.047**
DHEA (ng/dL)^a^	152.40 (96.60–205.50)	153.70 (91.27–214.35)	149.00 (124.70–195.00)	0.625
SHBG (nmol/L)^a^	41.00 (23.95–82.00)	48.50 (28.05–90.85)	27.00 (21.05–41.60)	**0.003**
hs-CRP (mg/L)^a^	1.20 (0.50–2.90)	1.05 (0.42–2.17)	2.70 (0.95–6.45)	**0.005**
Fasting insulin (uIU/mL)^a^	12.20 (9.15–15.80)	11.30 (8.70–13.07)	18.70 (16.75–21.85)	** < 0.001**
C-peptide (ng/mL)^a^	1.00 (0.75–1.60)	1.00 (0.70–1.20)	1.60 (1.45–1.95)	** < 0.001**
FBS (mg/dL)^a^	84.00 (80.50–92.00)	83.00 (79.25–88.00)	94.00 (86.5–102.00)	** < 0.001**
HbA1c (%)^a^	5.30 (5.10–5.50)	5.30 (5.00–5.50)	5.40 (5.15–50.50)	0.148
AST (U/L)^a^	18.00 (15.50–20.00)	18.00 (15.25–20.75)	18.00 (15.50–19.00)	0.970
ALT (U/L)^a^	10.00 (8.00–14.00)	9.50 (8.00–13.75)	12.00 (9.50–14.00)	0.064
Total cholesterol (mg/dL)^a^	171.00 (149.00–193.5)	167.5 (149.00–191.75)	180 (150.50–200.50)	0.1499
HDL- cholesterol (mg/dL)^a^	42.00 (34.00–48.00)	41 (34.00–49.50)	43.00 (36.00–47.00)	0.801
LDL-cholesterol (mg/dL)^a^	97.00 (82.00–114.00)	96 (81.00–110.75)	100.00 (83.50–122.00)	0.142
Triglycerides (mg/dL)^a^	106.00 (72.50–145.50)	100.50 (69.00–130.25)	141.00 (93.00–196.00)	**0.003**
Cr (mg/dL)^c^	0.80 ± 0.12	0.80 ± 0.12	0.80 ± 0.11	0.888
PCOM, yes^a^	104 (83.20)	79 (82.30)	25 (86.20)	0.621

Significant values are in bold.

Data are presented as median (IQR), mean ± SD or number (%).

^a^Mann–Whitney *U* test.

^b^Chi-squared test.

^c^Independent sample* t* test.

*HOMA-IR* homeostasis model assessment insulin resistance, *BMI* body mass index, *FFM* fat-free mass, *FSH* follicle-stimulating hormone, *LH* luteinizing hormone, *FAI* free androgen index, *DHEA* Dehydroepiandrosterone, *SHBG* Sex hormone-binding globulin, *hs-CRP* high-sensitivity C-reactive protein, *FBS* fasting blood sugar, *HbA1c* hemoglobin A1c, *AST* aspartate aminotransferase, *ALT* alanine aminotransferase, *HDL-C* high-density lipoprotein cholesterol, *LDL-C* low-density lipoprotein cholesterol, *Cr* creatinine, *PCOM* polycystic ovarian morphology.

The correlations of HOMA-IR with continuous variables are demonstrated in Table 2. HOMA-IR index levels were positively correlated with the BMI (r = 0.400, p < 0.001), and anthropometric measures including fat percentage (r = 0.437, p < 0.001), fat mass (r = 0.437, p < 0.001), FFM (r = 0.270, p = 0.002), and waist circumference (r = 0.418, p < 0.001) in PCOS women. However, no significant correlation was found between HOMA-IR index levels and age, acne score, hirsutism score, or systolic/diastolic blood pressure in study subjects. Among biochemical variables, we found that the HOMA-IR index was positively correlated with FAI (r = 0.286, p = 0.001), hs-CRP (r = 0.238, p = 0.008), triglycerides (r = 0.332, p < 0.001) and ALT (r = 0.260, p = 0.003); and was negatively correlated with SHBG (r = − 0.312, p < 0.001) in study participants. It is noteworthy that, despite predictable biochemical variables like fasting insulin, C-peptide and FBS, the HbA1c was not correlated with the HOMA-IR index. Despite the limited number of participants, we performed another correlation analysis stratified by BMI (Table 3), as the HOMA-IR was significantly correlated with BMI and anthropometric indices.

**Table 2:** Correlations of HOMA-IR with clinical and biochemical variables.

Variables	r coefficient	p-value
Age (years)	0.045	0.619
Acne score	0.037	0.681
Hirsutism score	− 0.034	0.707
BMI (kg/m^2^)	0.400	** < 0.001**
Fat percent (%)	0.437	** < 0.001**
Fat mass (kg)	0.437	** < 0.001**
FFM (kg)	0.270	**0.002**
Waist circumference (cm)	0.418	** < 0.001**
Systolic blood pressure (mmHg)	0.165	0.068
Diastolic blood pressure (mmHg)	0.145	0.108
FSH (mIU/mL)	0.043	0.632
LH (mIU/mL)	0.063	0.482
LH/FSH ratio	0.057	0.530
Testosterone (ng/mL)	0.051	0.569
FAI	0.286	**0.001**
DHEA (ng/dL)	− 0.018	0.841
SHBG (nmol/L)	− 0.312	** < 0.001**
hs-CRP (mg/L)	0.238	**0.008**
Fasting insulin (uIU/mL)	0.970	** < 0.001**
C-peptide (ng/mL)	0.512	** < 0.001**
FBS (mg/dL)	0.451	** < 0.001**
HbA1c (%)	0.130	0.153
AST (U/L)	0.125	0.166
ALT (U/L)	0.260	**0.003**
Total cholesterol (mg/dL)	0.125	0.165
HDL-cholesterol (mg/dL)	− 0.055	0.546
LDL-cholesterol (mg/dL)	0.114	0.206
Triglycerides (mg/dL)	0.332	** < 0.001**
Cr (mg/dL)	− 0.037	0.680

Significant values are in bold.

*HOMA-IR* homeostasis model assessment insulin resistance, *BMI* body mass index, *FSH* follicle-stimulating hormone, *LH* luteinizing hormone, *FAI* free androgen index, *DHEA* Dehydroepiandrosterone, *SHBG* Sex hormone-binding globulin, *hs-CRP* high-sensitivity C-reactive protein, *FBS* fasting blood sugar, *HbA1c* hemoglobin A1c, *AST* aspartate aminotransferase, *ALT* alanine aminotransferase, *HDL-C* high-density lipoprotein cholesterol, *LDL-C* low-density lipoprotein cholesterol, *Cr* creatinine.

**Table 3:** Correlations of HOMA-IR with clinical and biochemical variables categorized based on the BMI.

Variables	HOMA-IR			
	Normal weight (BMI < 25) (n = 48)		Overweight/obese (BMI ≥ 25) (n = 77)	
	R_s_ coefficient	p-value	R_s_ coefficient	p-value
Age (years)	− 0.065	0.663	-0.035	0.764
Acne score	0.027	0.856	0.084	0.466
Hirsutism score	0.058	0.696	-0.013	0.911
Systolic blood pressure (mmHg)	− 0.101	0.493	0.130	0.263
Diastolic blood pressure (mmHg)	− 0.357	**0.013**	0.239	**0.038**
FSH (mIU/mL)	0.108	0.464	0.009	0.936
LH (mIU/mL)	0.069	0.641	0.091	0.431
LH/FSH ratio	0.056	0.706	0.081	0.483
Testosterone (ng/mL)	0.102	0.488	0.001	0.991
FAI	0.150	0.314	0.303	**0.007**
DHEA (ng/dL)	0.050	0.738	0.037	0.747
SHBG (nmol/L)	− 0.120	0.418	− 0.372	**0.001**
hs-CRP (mg/L)	0.217	0.139	0.159	0.168
Fasting insulin (uIU/mL)	0.975	** < 0.001**	0.958	** < 0.001**
C-peptide (ng/mL)	0.271	0.063	0.551	** < 0.001**
FBS (mg/dL)	0.376	**0.008**	0.499	** < 0.001**
HbA1c (%)	0.085	0.567	0.141	0.228
AST (U/L)	0.215	0.142	0.010	0.929
ALT (U/L)	0.290	**0.046**	0.173	0.132
Total cholesterol (mg/dL)	− 0.079	0.591	0.199	0.083
HDL-cholesterol (mg/dL)	− 0.188	0.200	0.114	0.323
LDL-cholesterol (mg/dL)	− 0.107	0.470	0.181	0.116
Triglycerides (mg/dL)	0.264	0.070	0.330	**0.003**
Cr (mg/dL)	0.046	0.755	− 0.079	0.493

Significant values are in bold.

*HOMA-IR* homeostasis model assessment insulin resistance, *BMI* body mass index, *FSH* follicle-stimulating hormone, *LH* luteinizing hormone, *FAI* free androgen index, *DHEA* Dehydroepiandrosterone, *SHBG* Sex hormone-binding globulin, *hs-CRP* high-sensitivity C-reactive protein, *FBS* fasting blood sugar, *HbA1c* hemoglobin A1c, *AST* aspartate aminotransferase, *ALT* alanine aminotransferase, *HDL-C* high-density lipoprotein cholesterol, *LDL-C* low-density lipoprotein cholesterol, *Cr* creatinine.

Analysis of data from normal weight participants revealed that HOMA-IR index levels were positively correlated with the fasting insulin (r = 0.975, p < 0.001), FBS (r = 0.376, p = 0.008), and ALT (r = 0.290, p = 0.046); it was also negatively correlated with diastolic blood pressure (r = − 0.357, p = 0.013).

In overweight/obese participants, the HOMA-IR index was positively correlated with fasting insulin (r = 0.958, p < 0.001), FBS (r = 0.499, p < 0.001), C-peptide (r = 0.551, p < 0.001), FAI (r = 0.303, p = 0.007), triglycerides (r = 0.330, p = 0.003) and diastolic blood pressure (r = 0.239, p = 0.038); and was negatively correlated with SHBG (r = − 0.372, p = 0.001).

Clinical and laboratory parameters have been compared between various PCOS phenotype groups (Table 4). Among a total of 125 PCOS participants, the O–H–P phenotype (56%) was the most prevalent, followed by O–H (16.8%) and H–P (16.8%), as well as O–P (10.4%) phenotypes. No difference between the four groups was recorded with respect to demographic, clinical, and biochemical characteristics, except for FBS levels (p = 0.038), which was the highest among O–H–P phenotype. In addition, in the Bonferroni post hoc test, no significant difference was found between each two groups.

**Table 4:** Distribution of clinical and biochemical features among PCOS phenotypes.

Variables	H–P (n = 21)	O–H (n = 21)	O–P (n = 13)	O–H–P (n = 70)	p-value
Age (years)^a^	28.00 (25.00–34.00)	26.00 (20.50–32.00)	30.00 (23.00–34.00)	28.00 (21.75–32.00)	0.438
BMI (kg/m^2^)^b^	26.05 ± 4.64	24.69 ± 4.69	26.52 ± 5.97	28.34 ± 6.34	0.061
Fat percent (%)^a^	33.4 (30.75–37.20)	35.30 (29.10–39.35)	30.40 (27.20–42.75)	37.50 (32.35–42.60)	0.104
Fat mass (kg)^a^	22.00 (18.20–25.60)	22.10 (15.85–28.90)	19.40 (14.97–32.70)	27.70 (19.75–34.30)	0.114
FFM (kg)^a^	44.40 (40.00–47.30)	41.1 (39.10–44.35)	42.20 (40.17–44.17)	43.20 (40.75–47.40)	0.181
Waist circumference (cm)^a^	88.00 (78.00–98.25)	89.00 (78.00–97.50)	91.75 (89.00–96.50)	94.00 (84.50–103.00)	0.215
Systolic blood pressure (mmHg)^b^	103.81 ± 16.42	108.09 ± 12.50	101.92 ± 11.28	108.29 ± 14.96	0.358
Diastolic blood pressure (mmHg)^b^	68.09 ± 14.62	75.00 ± 10.25	70.77 ± 8.62	74.93 ± 10.27	0.064
FSH (mIU/mL)^a^	6.20 (4.25–7.35)	4.80 (2.75–5.90)	5.70 (4.20–7.15)	5.70 (4.27–6.62)	0.307
LH (mIU/mL)^a^	8.40 (4.20–14.60)	4.60 (2.10–14.95)	9.10 (3.25–17.15)	11.00 (6.40–19.62)	0.083
LH/FSH ratio^a^	1.68 (0.92–2.32)	1.00 (0.58–2.45)	1.75 (0.66–2.80)	2.06 (1.14–3.52)	0.173
hs-CRP (mg/L)^a^	1.30 (0.40–1.75)	0.90 (0.20–4.00)	0.70 (0.30–2.50)	1.50 (0.67–3.22)	0.147
HOMA-IR^b^	2.55 (1.78–3.03)	2.39 (1.51–3.28)	2.75 (2.34–2.89)	2.65 (1.89–3.65)	0.438
Fasting insulin (uIU/mL)^a^	12.30 (8.90–15.15)	11.70 (7.20–16.05)	12.10 (11.15–13.50)	12.25 (9.40–16.45)	0.794
C-peptide (ng/mL)^a^	0.90 (0.60–1.55)	0.55 (1.00–1.20)	1.00 (0.65–1.25)	1.10 (0.80–1.60)	0.169
FBS (mg/dL)^a, c^	83.00 (76.50–89.00)	82.00 (77.50–89.00)	85.00 (83.00–94.50)	86.00 (81.00–95.00)	**0.038**
HbA1c (%)^a^	4.92 (5.40–5.57)	5.30 (5.00–5.45)	5.20 (4.92–5.37)	5.30 (5.20–5.50)	0.394
AST (U/L)^a^	17.00 (16.00–20.50)	17.00 (15.00–19.00)	18.00 (16.00–20.00)	17.50 (15.00–21.00)	0.903
ALT (U/L)^a^	11.00 (9.50–14.50)	9.00 (7.00–14.00)	11.00 (7.00–13.50)	10.00 (8.00–13.25)	0.586
Total cholesterol (mg/dL)^a^	177.00 (161.50–191.00)	165.00 (142.50–200.00)	166.00 (131.50–189.00)	171.00 (149.00–194.00)	0.777
HDL-cholesterol (mg/dL)^a^	40.00 (34.50–48.50)	40.00 (32.50–49.50)	42.00 (35.00–43.50)	44 (34.00–50.00)	0.619
LDL-cholesterol (mg/dL)^a^	100.00 (89.00–109.50)	86.00 (75.50–122.50)	96.00 (76.00–114.5)	96.50 (82.00–115.00)	0.654
Triglycerides (mg/dL)^a^	88.00 (69.00–138.50)	99.00 (67.00–141.50)	104.00 (62.50–156.50)	110.00 (75.50–148.00)	0.591
Cr (mg/dL)^b^	0.80 ± 0.13	0.81 ± 0.14	0.81 ± 0.15	0.79 ± 0.10	0.867

Significant values are in bold.

Data are presented as median (IQR) or mean ± SD.

^a^Kruskal–Wallis.

^b^One-way analysis of variance (ANOVA).

^c^The differences between each two groups was not significant (p > 0.05) using Bonferroni post hoc test.

*H* hyperandrogenism, *O* oligomenorrhea, *P* polycystic ovarian morphology, *BMI* body mass index, *FFM* fat-free mass, *FSH* follicle-stimulating hormone, *LH* luteinizing hormone, *hs-CRP* high-sensitivity C-reactive protein, *HOMA-IR* homeostasis model assessment insulin resistance, *FBS* fasting blood sugar, *HbA1c* hemoglobin A1c, *AST* aspartate aminotransferase, *ALT* alanine aminotransferase, *HDL-C* high-density lipoprotein cholesterol, *LDL-C* low-density lipoprotein cholesterol, *Cr* creatinine.

In logistic regression analysis using insulin resistance (HOMA-IR ≥ 3.46) as a dependent variable, phenotype O–H–P (odds ratio [OR] = 2.52, 95% confidence interval [CI] 1.02–6.24) was shown to be associated with about two-fold increased risk of insulin resistance (p < 0.05) as shown in Table 5.

**Table 5:** Association between PCOS phenotypes with insulin resistance (HOMA-IR ≥ 3.46) in logistic regression analysis (sample size n = 125).

Variables^a^	OR	95%CI	p-value
H–P	2.08	0.72–5.98	0.175
O–H	2.84	0.91–8.88	0.073
O–P	1.80	0.70–4.65	0.22
O–H–P	2.52	1.02–6.24	**0.046**

Significant values are in bold.

*H* hyperandrogenism, *O* oligomenorrhea, *P* polycystic ovarian morphology, *OR* odds ratio, *CI* confidence interval.

^a^Variables included into the multivariate model were age, H–P, O–H, O–P, and O–H–P using the backward selection approach.

## Discussion

In this cross-sectional analysis, abnormalities of anthropometric parameters, higher triglyceride levels, hs-CRP, ALT, and FAI as well as lower SHBG were observed among insulin-resistant women with PCOS.

We noticed higher hs-CRP levels in participants with abnormal HOMA-IR values. In recent years, increasing attention has been paid to the significance of inflammation in PCOS. Interleukin-18, as a potent proinflammatory biomarker, was shown to be related to indices of adiposity and insulin resistance in PCOS^19^. In response to hyperglycemia, mononuclear cells produce tumor necrosis factor-α (TNF-α), which can exacerbate the metabolic abnormalities of PCOS^20^. Adipose tissue-resident macrophages release interleukin-6, which is contributed to insulin resistance in PCOS^21^. A systematic review and meta-analysis of 63 studies found that women with PCOS had significantly higher circulating CRP levels than controls (standardized mean difference 1.26, 95% CI 0.99–1.53). However, a high heterogeneity among studies was found^22^. The inflammatory state in PCOS is thought to interact with hyperinsulinemia, insulin resistance, and obesity^23^. Preliminary data indicate that application of insulin-sensitizing agent therapy may decrease the inflammatory state in PCOS^24,25^. Serum hs-CRP was shown to be positively correlated with serum insulin, insulin resistance, fat mass, and body weight in women with PCOS^26,27^. Our analyses suggest that the inflammation is due to PCOS-related insulin resistance rather than PCOS itself; but further evidence is required to support the mechanisms of inflammation and the role of insulin in PCOS.

Our study also found an association between FAI and SHBG levels with the HOMA-IR index. Previous studies suggested that low SHBG levels in the general population are a predictor of increased risk of developing type 2 diabetes mellitus^28^, hypertension^29^, and metabolic syndrome^30^. We also found a weak negative correlation between HOMA-IR and SHBG in PCOS overweight/obese women. A moderate correlation was observed in another study in PCOS women (r = − 0.557, p < 0.001)^31^. Compensatory hyperinsulinemia as a result of insulin resistance can suppress the synthesis of SHBG in the liver^32^. In addition, the relationship between low serum SHBG and impaired glucose metabolism can be explained by the hypothesis that the phosphatidylinositol 3-kinase (PI3K)/protein kinase B (AKT) signaling pathway is regulated by SHBG. Activation of upstream PI3K may cause phosphorylation of Mammalian target of rapamycin (mTOR), and consecutive insulin resistance^32^. Noteworthy, another study indicated that the association between glycemic parameters and SHBG depends on BMI, and SHBG is not reflective of insulin resistance in PCOS women^33^. In a similar way, the observed correlations between FAI or SHBG and HOMA-IR in overweight/obese PCOS women were not evident in normal weight PCOS women in our study.

We found a positive correlation between HOMA-IR and diastolic blood pressure in obese/overweight PCOS women, which is consistent with a previous study that indicated higher diastolic blood pressure in obese hyperinsulinemic PCOS women compared to lean hyperinsulinemic ones^34^. Higher diastolic pressure was also reported among obese PCOS women compared with non-obese subjects^35–37^. Additionally, we found an opposite weak correlation between HOMA-IR and diastolic blood pressure in normal weight PCOS women. It might be a potential source of uncertainty in our study and it is difficult to extrapolate our results to other PCOS women.

Our analyses also showed a weak positive correlation between HOMA-IR and ALT levels in normal weight participants. It is to be noted that we did not screen participants regarding non-alcoholic fatty liver disease (NAFLD). Nevertheless, elevated liver enzymes, as a surrogate marker of NAFLD, are common in women with PCOS^38,39^. A recent meta-analysis reported that higher values of HOMA-IR, FAI, ALT, and triglycerides, as well as obesity were all associated with significantly higher risk-adjusted odds of NAFLD in PCOS women^40^. It was also shown that the HOMA-IR indexes in the PCOS group complicated with NAFLD were higher than those in the control group complicated with NAFLD^41^.

There is evidence of high ALT levels in lean PCOS patients^42^. Our study suggests that ALT levels in PCOS may be linked to insulin resistance, irrespective of obesity. The possible explanation is that in young lean insulin-sensitive subjects, energy is mainly stored in the liver and muscle glycogen. However, in lean insulin-resistant subjects, energy is commonly diverted to the liver triglyceride synthesis, causing hepatic steatosis and NAFLD in PCOS^43^. Nevertheless, more evidence is required to confirm such associations. We also made a comparison among different PCOS phenotypes. We found no difference among the four phenotypes of PCOS regarding demographic, clinical, and biochemical characteristics, except for FBS levels. FBS levels were highest among PCOS women with O–H–P phenotype. Conflicting results are reported on the blood sugar status in the PCOS phenotypes from different ethnic groups. Contrary to the results of our study, several researchers did not show differences in blood glucose levels between PCOS phenotypes^44–46^, while there is also evidence of higher FBS in O–H–P phenotype^47–49^. It remains to be elucidated whether blood sugar status differs among PCOS phenotypes.

The association of HOMA-IR in PCOS phenotypes is variable across the studies. Some previous studies have investigated the risk of insulin resistance in the various PCOS phenotypes and showed that HOMA-IR does not differ among the four PCOS phenotypes^50,51^, while the results of some other studies indicated a higher risk of insulin resistance in the phenotype O–H–P^52–55^. In this study, we showed a higher risk of insulin resistance in the classic PCOS phenotype. This disagreement may be due to different study methodologies, defining criteria for insulin resistance, cut-off values for HOMA-IR, or PCOS classification criteria.

This study has its strengths; a comprehensive comparison of clinical and biochemical profiles of PCOS women with/without insulin resistance and also among different PCOS phenotypes was provided. Phenotypic categorization of PCOS women can help to predict risk of insulin resistance. To mention the limitations, the current study was restricted to Iranian women, and further research is required with a larger sample size involving patients of different ethnicities. Another limitation of the current study is the use of HOMA-IR cut-off for diagnosis of insulin resistance. However, the euglycemic hyperinsulinemic clamp is the gold standard method for direct measurement of insulin resistance.

## Conclusion

In conclusion, the results of this study revealed that PCOS women diagnosed with insulin resistance based on HOMA-IR should be monitored in regard to visceral obesity, blood pressure, liver enzymes, and hypertriglyceridemia. Moreover, phenotype division may help physicians to predict adverse metabolic outcomes. The results of this study suggest that full-blown PCOS (O–H–P) women have an increased risk of insulin resistance and may need routine screening for metabolic disturbances.

## Data Availability

The datasets used and/or analyzed during the current study are available from the corresponding author upon reasonable request.
